# Lateral epitaxial heterojunctions in single nanowires fabricated by masked cation exchange

**DOI:** 10.1038/s41467-018-02878-w

**Published:** 2018-02-06

**Authors:** Sedat Dogan, Stefan Kudera, Zhiya Dang, Francisco Palazon, Urko Petralanda, Sergey Artyukhin, Luca De Trizio, Liberato Manna, Roman Krahne

**Affiliations:** Istituto Italiano di Tecnologia, Via Morego 30, 16163 Genova, Italy

## Abstract

Cation exchange is a versatile tool to control the composition of nanocrystals, and recently deterministic patterning could be achieved by combining it with lithography techniques. Regarding single nanocrystal structures, such spatial control of cation exchange enables the design of heterostructures, which can be integrated in functional optoelectronic elements. In this work, we fabricate nanowire CdSe/Cu_2_Se heterojunctions by masking cation exchange via electron-beam irradiation, such that cation exchange proceeds only in the non-irradiated sections. Interestingly, the heterojunction interfaces are almost atomically sharp, and the adjacent CdSe and Cu_2_Se domains exhibit epitaxial relationships. We show that the cation exchange at the CdSe/Cu_2_Se interface is only possible if the displaced Cd^2+^ ions can radially out-diffuse to the solution phase. If this exit pathway is blocked, the cation exchange cannot occur. Our technique allows one to transform already contacted single nanowires, and the obtained heterojunction nanowires manifest a noticeable gain in conductance.

## Abstract (web summary)

Lateral structuring of the chemical composition of nanowires can enhance their functionality for photoconductive devices. Here, the authors fabricate CdSe/Cu_2_Se nanowire heterojunctions with atomically sharp interfaces and epitaxial relationships by a masked cation exchange approach.

## Introduction

Ion-exchange reactions applied to nanomaterials are extremely useful tools for fabricating nanostructures with control over size, shape, and chemical composition^1–5^. Cation exchange (CE) reactions, for example, are now routinely used to completely or partially replace the cations of preformed nanocrystals with different types of cations, retaining their size and shape^6^. While the total replacement of native cations can lead to the formation of new nanocrystal materials^7^, sometimes even with metastable crystal structures^8,9^, partial CE reactions allow for the fabrication of nano-heterostructures^10–12^ with well-defined interfaces^13–15^ in architectures ranging from core-shell^16,17^ to striped^18–20^ or Janus-like^2^. This technique, initially developed for metal chalcogenides, has been extended to III–V semiconductors^21–24^ and, more recently, to the emerging halide perovskite materials^25–28^, for which both cations and anions can be effectively exchanged^29,30^.

Recently, we discovered that ion-exchange reactions in nanocrystal films can be blocked by the irradiation of the film with a high-energy electron or X-ray beam^31,32^. Secondary electrons generated by such high-energy primary beams partially degrade the ligand shell around the nanocrystals and create a barrier that shields the nanocrystals from the inflow and outflow of ions. By designing tailored exposure patterns, we could also fabricate laterally structured nanocrystal films with different chemical compositions, i.e., also with different optical, electrical, or mechanical properties^33^. The possibility to apply this masking procedure to a single crystalline nanostructure (for example, a sheet, wire, or any other nanocrystal shape) raises many exciting opportunities and questions. The main question is whether or not the desired pattern can be successfully transferred in single crystal nanostructures since, in principle, ion diffusion could be isotropic and occur in non-desired directions, causing exchange also in the masked regions. Masking nanowires (NWs) with conventional photoresist lithography has indeed succeeded in protecting the resist-covered regions from ion-exchange^16,34^, but a detailed understanding of the masked cation exchange process in single nanostructures is still lacking.

Here we consider the CdSe → Cu_2_Se exchange reaction for our study and show that electron beam irradiation of sections of surfactant-coated CdSe NWs is as effective as conventional photoresist lithography in preventing cation exchange (CE) reactions with Cu^+^ ions in the irradiated regions: the exchange is complete in the non-irradiated regions, transforming them into Cu_2_Se, but it does not proceed into the irradiated sections, such that epitaxial CdSe/Cu_2_Se junctions are formed. Furthermore, we apply the masked CE process to single NWs already contacted by electrodes, a setting that enables us to measure the conductivity of the individual devices before and after the exchange. We find that the partial conversion of the NW between the two electrodes can enhance its dark and photoconductance by several orders of magnitude.

## Results

### Masked cation exchange process

**Fig. 1:**
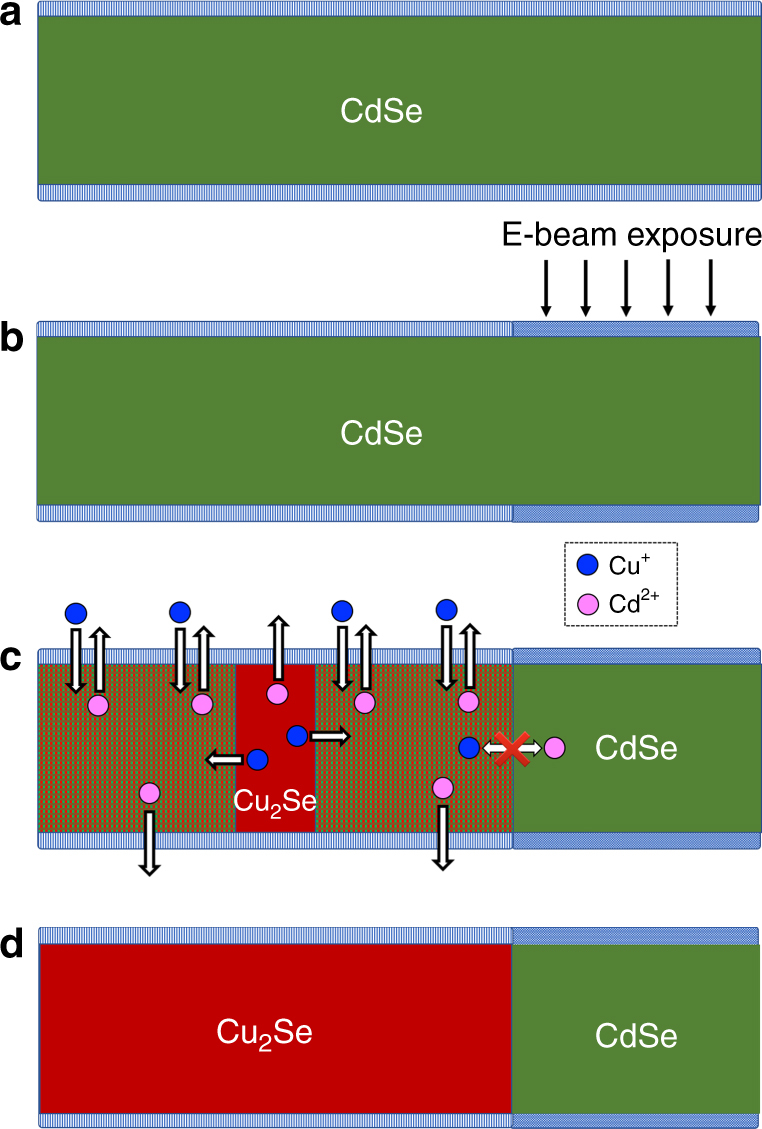
Illustration of the masked cation-exchange process in single CdSe nanowires. The layer of surface ligands of a pristine CdSe NW (**a**) gets locally exposed to an electron beam that renders it impenetrable for cations (**b**). The CE reaction initiates in the unexposed regions and proceeds laterally until it reaches the interface with the e-beam irradiated region (**c**), which results in a heterojunction with an epitaxial sharpness (**d**). Cu^+^ (Cd^2+^) cations are sketched by blue (magenta) circles, and the arrows illustrate the diffusion directions

The formation of the CdSe/Cu_2_Se heterostructures is illustrated in Fig. 1. It can be rationalized by considering first that the CE reaction can only start in the non-irradiated regions of the wire, as these are the only regions accessible to the Cu^+^ ions, with the displaced Cd^2+^ ions being expelled out to the liquid phase. Since CdSe and Cu_2_Se are immiscible and the wires have a thickness of few tens of nm, a Cu_2_Se wire section is quickly formed and expands over time, with CdSe regions being transformed to Cu_2_Se (see Fig. 1c). In principle, the exchange could continue also in the irradiated region of the wire, if Cu^+^ ions were to diffuse axially from the Cu_2_Se section into the CdSe one and the displaced Cd^2+^ ions were to follow the opposite diffusive path (from CdSe to Cu_2_Se). This would be, in fact, the only way for Cd^2+^ ions to escape from the irradiated CdSe regions, since their radial out-diffusion through CdSe is blocked by the layer of cross-linked molecules on the surface of the wire. However, our calculations indicate that, while interstitial Cu^+^ ions can easily diffuse through CdSe, the exchange of interstitial Cd^2+^ ions with Cu^+^ ones at the interface requires a considerable amount of energy. Therefore, cation diffusion across the interface is severely hindered (this is indicated by the crossed-out arrow in Fig. 1c). Hence, CE does not occur in the irradiated regions because Cd^2+^ ions cannot escape from there, neither radially via the surface of the wire, nor axially through the Cu_2_Se domain.

### Cation exchange and transmission electron microscopy analysis

**Fig. 2:**
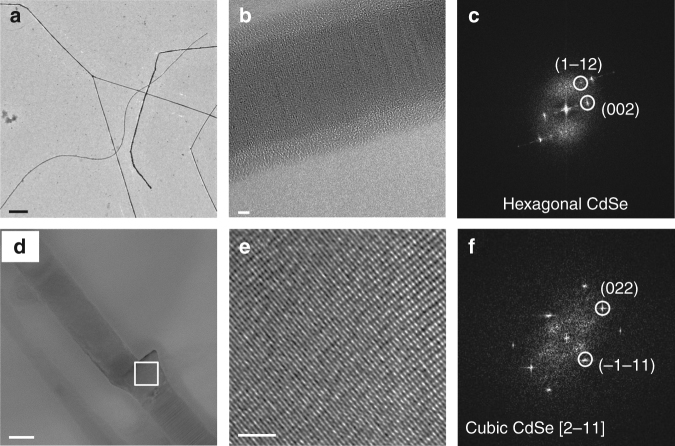
Transmission electron microscopy analysis. **a** Low-magnification TEM image of the CdSe NWs fabricated by seeded solution synthesis. Scale bar is 1 µm. **b** HRTEM image of the CdSe NWs showing defects in crystal lattice structure. Scale bar is 2 nm. **c** Corresponding FFT demonstrating that the wire is oriented along the [110] zone axis, and elongated in the < 001 > direction of the wurtzite structure (ICSD: 415784). **d** HRTEM image of the CdSe/Cu_2_Se heterojunction, scale bar is 20 nm. **e** Magnified view of the CdSe region marked with a box in **d**, showing that the defects were eliminated after electron irradiation. Scale bar is 2 nm. A detailed analysis of defects in irradiated and non-irradiated regions is presented in the Supplementary Figure 1. **f** The corresponding FFT showing the cubic crystal structure of CdSe (ICSD: 53954)

Figure 2a shows a low-resolution transmission electron microscopy (TEM) image of the as-synthesized CdSe NWs that have diameters ranging from 30 to 80 nm and lengths exceeding 10 µm. High-resolution TEM (HRTEM) combined with Fast-Fourier-Transform (FFT) analysis (Fig. 2b, c) reveals an overall wurtzite lattice structure (ICSD: 415784) with the NW elongated in <001> direction, albeit with a large quantity of stacking faults. We apply the masked CE process to single NWs that were previously deposited on different substrates, namely ultrathin holey carbon coated Au grids for HRTEM analysis, and Si/SiO_2_ wafers for electrical measurements. The concept of the procedure is illustrated in Fig. 1. After locating the NW with swift and low-dose scanning electron microscopy of 71 µC/cm^2^ (below the threshold to inhibit CE as previously shown^31^), one section of the wire is exposed to an electron beam at a high dose of 400 mC/cm^2^, which converts the ligands at the NW surface into a layer that is impermeable to cations^31^. HRTEM analysis of the same wire before and after irradiation reveals that the irradiation results in a phase transition from the hexagonal wurtzite to the cubic sphalerite phase (ICSD: 53954), which is accompanied by a significant reduction in the number of stacking faults (as can be seen in Fig. 2d, e, and which is discussed in more detail in the Supplementary Notes 1).

**Fig. 3:**
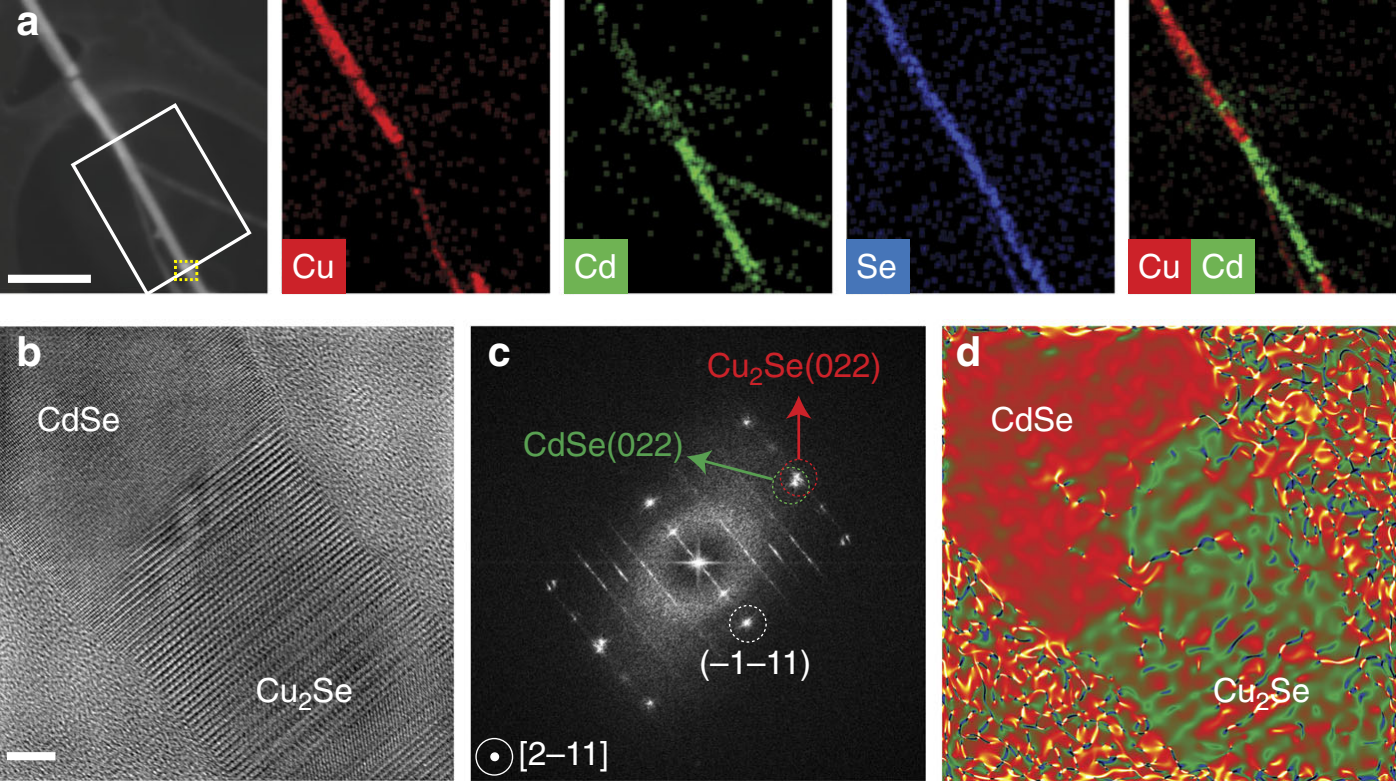
Compositional and crystallographic analysis of nanowire heterojunctions. **a** HAADF-STEM image, and the corresponding STEM-EDS elemental maps, of a heterojunction CdSe/Cu_2_Se NW. The white box indicates the region that was exposed to e-beam irradiation. Scale bar is 200 nm. **b** HRTEM image of the CdSe/Cu_2_Se interface found in the heterojunction’s region marked with a dashed yellow box in **a**, with the corresponding FFT showing the epitaxial relationship between CdSe and Cu_2_Se (**c**). **d** Mean dilation map calculated from the GPA analysis of the HRTEM image shown in **b**. Scale bar in **b** is 5 nm

Subsequently, the sample is submerged in a solution of Cu^+^ cations in methanol (4 mL, [Cu^+^] = 75 mg/mL) for 5 min at room temperature to trigger the CdSe→Cu_2_Se CE transformation. The CE reaction is stopped in clean methanol, and then the samples are dried under N_2_ flow. Fig. 3a displays a representative high-angle annular dark-field scanning TEM (HAADF-STEM) image of a single NW (same one as shown in Fig. 2d) with a diameter of around 40 nm, in which the region marked with a white box in panel (a) has undergone masked CE. More specifically, the lower half of the NW in the boxed-region had been exposed to the electron-beam before the CE step. The content of Cu (red) and Cd (green) in the STEM-energy dispersive X-ray spectroscopy (EDS) maps (Fig. 3a) indicates that the exchange with Cu^+^ ions has only occurred in the upper part of the NW, while the lower part has retained the original composition. Furthermore, as the Se content (blue) is evenly distributed through the whole wire, we can conclude that the anion sublattice is maintained. Interestingly, the newly formed CdSe/Cu_2_Se interface appears atomically sharp in HRTEM images (see Fig. 3b). The two sections of the junction possess an epitaxial relationship: CdSe [2–11] // Cu_2_Se [2–11], CdSe (011) // Cu_2_Se (011), as seen in the corresponding FFT images (Fig. 3c). The lattice mismatch between cubic CdSe and the newly formed cubic Cu_2_Se (ICSD: 67050) is consistent with the −3.9% relative dilatation (in the green relative to the red area) estimated by geometric phase analysis (GPA) of the HRTEM image in Fig. 3d. Indeed, a lattice contraction of 3.8% is expected when going from cubic CdSe to cubic Cu_2_Se as the lattice parameters of these materials are 6.05 Å and 5.83 Å, respectively. Also, while the unexchanged cubic CdSe section of the NW remains relatively free from defects, the exchanged Cu_2_Se section is characterized by a high density of stacking faults, which are, most likely, inherited from the original, highly defective CdSe wurtzite section (see other heterojunctions in Supplementary Figure 2).

We have also performed partial cation exchange experiments, using substoichiometric amounts of Cu^+^ ions, in order to obtain information about the initial stages of the CE process (see “Methods” section and Supplementary Notes 2). In this regime, we observe that either small segments of the wire are completely exchanged, or otherwise a small amount of Cu is distributed all along the wires, while the Cd signal is still strongly present (see Supplementary Figure 3). This supports the mechanism that we propose in Fig. 1, with the CE progressing swiftly along the wire once a segment is transformed.

Also, we note that extended exposure (several hours) of our heterostructures to air oxidizes the Cu_2_Se domains into Cu_2−*x*_Se ones, that is, they become substoichiometric in Cu. This is confirmed by X-ray Photoelectron Spectroscopy (XPS) and X-ray-excited Auger Electron Spectroscopy (XAES) analysis (see Supplementary Notes 3 with its Supplementary Figures 4 and 5) and is a well-known effect in copper chalcogenides ^35–37^.

### Computational modeling

**Fig. 4:**
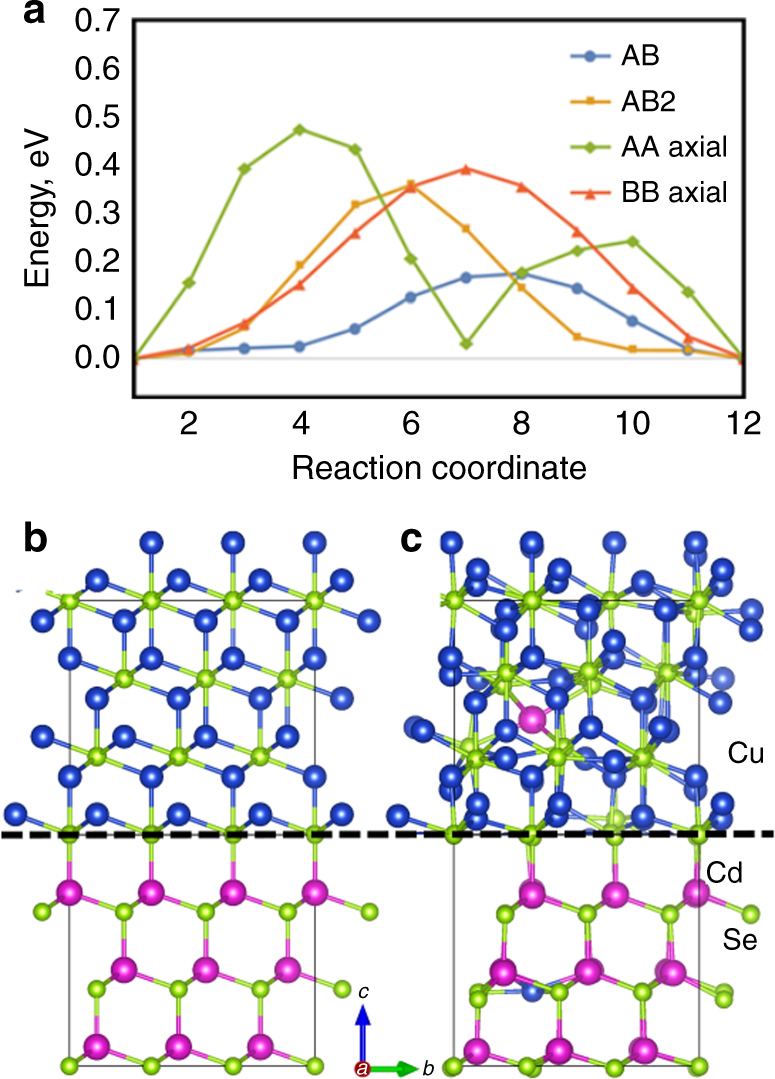
Computational modeling. **a** Energy barriers along the Cu^+^ diffusion paths in wurtzite CdSe structure, as calculated using the NEB method (see also Supplementary Figure 6c). The radial diffusion is dominated by successive AB steps, while the axial diffusion is facilitated by alternating AB and AB2 steps. **b**, **c** Scheme of the CdSe–Cu_2_Se interface (illustrated with a dashed line) with antifluorite Cu_2_Se above and zinc blende CdSe below. The structures with defects were obtained by structural optimization and then brought together to illustrate the stacking

We have also investigated the Cu-for-Cd CE substitution in the NWs by computational modeling. Here we first focus on the processes in the non-irradiated sections and we study the Cu^+^ diffusion process in the hexagonal wurtzite (space group P6_3_mc) CdSe using the Nudged Elastic Band (NEB) method^38,39^ (see computational details in the “Methods” section and the Supplementary Notes 4). We calculate the diffusion barriers of Cu^+^ cations along the (*a, b)* and *c* directions of the wurtzite lattice, which correspond to the radial and axial directions of the wire, respectively. We identify two interstitial equilibrium positions, A and B, for the impurities, as shown in Supplementary Figure 6. Our calculations show that the Cu^+^ diffusion occurs roughly 8 times faster in the radial than in the axial direction (see Fig. 4a for plots of the diffusion barriers along the possible reaction pathways, and the Supplementary Information section with Supplementary Figure 6 for more details). Next, we consider the processes at the interface between the irradiated and non-irradiated regions, assuming a total Cu_2_Se conversion of the non-irradiated part. According to our HRTEM results, we consider that CdSe in the irradiated region has a cubic phase, with an epitaxial interface to Cu_2_Se. In detail, we model the interface by a box containing antifluorite (space group Fm-3m) Cu_2_Se and sphalerite (space group F-43m) CdSe, stacked in the <111> direction (see Fig. 4b, c). In order for the cation exchange to proceed, cadmium ions have to diffuse into Cu_2_Se, while copper ions into CdSe. Consequently, the CdSe section acquires a Cd vacancy and a Cu interstitial defect, while a Cu vacancy and Cd interstitial are formed in Cu_2_Se section. We therefore estimate the energy barrier for this process by computing the difference between the total energies of the defected supercells of CdSe and Cu_2_Se and the ideal supercells. This results in a barrier of around 2 eV, prohibitive at a room temperature. Therefore, we conclude that the diffusion of Cu^+^ cations into the (irradiated) CdSe region is energetically very expensive, since it needs to be balanced by Cd^2+^ cations moving into the Cu_2_Se section (the Cd^2+^ cations cannot escape radially in the irradiated region due to the blocking layer at the surface).

## Discussion

On the basis of our calculations, we can present the following model for the masked CE process on single CdSe NWs, as already sketched in Fig. 1: At the initial stage of the CE reaction, Cu^+^ ions access unmasked regions of the NW and replace Cd^2+^ ions, mainly by radial diffusion from the surface, since this is the more effective diffusion pathway. Once the Cu^+^-for-Cd^2+^ exchange is completed in the unmasked regions, the CE process cannot proceed into the masked CdSe domains for the following reasons: (i) the cross-linked ligands surrounding the CdSe masked sections of the NWs behave as a blocking layer for the inflow and outflow of cations and, consequently, Cu^+^ and Cd^2+^ ions cannot access or escape the NW from the lateral sides; (ii) Cu^+^ ions cannot diffuse from the Cu_2_Se into the CdSe section to replace Cd^2+^ ions in the latter due to the high cost in energy of interstitial Cd^2+^ ions in Cu_2_Se. This finding is consistent with what we had observed in a previous work, in which stoichiometric Cu_2_Se NCs were exposed to Cd^2+^ ions at room temperature^40^. In that case, the diffusion of Cd^2+^ ions inside the host NCs was extremely slow, resulting in almost no exchange even after prolonged reaction times^40^. We tend to exclude any role of the cubic CdSe crystal structure in preventing the diffusion of Cu^+^ ions across the interface because there are low-energy diffusion pathways for Cu^+^ ions in cubic CdSe (see Supplementary Figures 9 and 7), thus the cubic CdSe phase itself is not preventing the entrance of Cu^+^ ions.

**Fig. 5:**
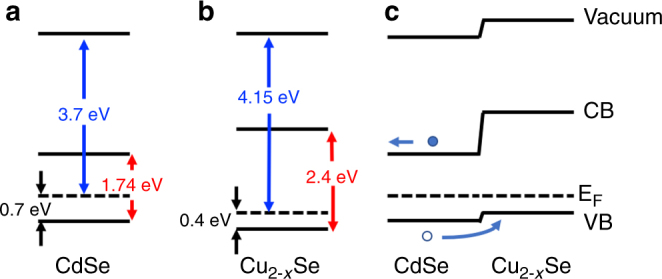
Flat band schemes. Energy levels of the CdSe (**a**), Cu_2−*x*_Se (**b**), and of the heterojunction (**c**) as obtained from ultraviolet photoelectron spectroscopy (UPS) data (see Supplementary Figure 8) and the bulk band gap values. Energies of Fermi level to vacuum are stated in blue, valence band to Fermi level in black, and the band gap in red color. The full and hollow circles in **c** represent the photoexcited electrons and holes, respectively, and the arrows indicate the charge separation induced by the heterojunction

We have performed ultraviolet photoelectron spectroscopy (UPS) on CdSe and the cation-exchanged (and oxidized) Cu_2−*x*_Se nanowires to gain insight in their energy level structure, and on the energy level alignment at the heterojunction. This technique allows to determine the energy difference between vacuum and Fermi level, and between the top of the valence band and Fermi level (see Supplementary Notes 5). Fig. 5 shows the energy levels based on the values that we obtained by UPS, and by considering the bulk band gap of CdSe^41^ and Cu_2−*x*_Se^37^. The Fermi-level to valence band offsets are in good agreement with what we would expect for the undoped CdSe nanowires and for the Cu deficient Cu_2−*x*_Se material (that is known to show a plasmon resonance from free holes^37^). The resulting heterojunction, sketched in Fig. 5c, should translate into an asymmetric current–voltage curve, with higher conductivity when the CdSe section is under positive bias. Also, the photocurrent, arising from the photoexcited carriers which are generated in the CdSe section, should be favored under this condition.

**Fig. 6:**
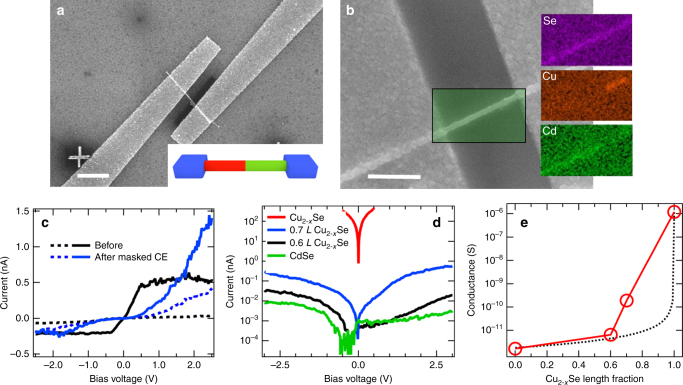
Electrical properties of single wires. **a** SEM image of a single NW contacted with two aluminum electrodes. The inset illustrates the heterojunction NW that is obtained after masked CE. Scale bar is 2 µm. **b** SEM image with higher resolution and EDS maps showing the Se, Cu, and Cd content of the wire section between the electrical contacts after CE. Scale bar is 500 nm. **c** Dark and photo IV characteristics of a single CdSe NW before and after the masked CE that converted it into a CdSe/Cu_2−*x*_Se heterojunction NW. The samples were illuminated with white light at 35 mW/cm^2^. **d** Current–voltage curves in the dark of a CdSe NW, two heterojunction NWs with 0.6*L* and 0.7*L* Cu_2−*x*_Se length fraction (where *L* is the NW length in between the electrodes), and of a fully converted Cu_2−*x*_Se NW. Due to its higher conductance, the Cu_2−*x*_Se NW was measured only up to +/− 0.5 V to avoid damage. Linear plots are shown in Supplementary Figure 10. **e** NW conductance vs. relative length of the Cu_2−*x*_Se section obtained at a bias voltage of −3 V, for a wire with diameter of 80 nm and *L* = 730 nm. The dotted line shows the conductance that would result from two ohmic resistors in series

To study the changes in the electrical properties induced by the CE and to explore the usefulness of such heterojunction NWs in electrical devices, we have performed measurements on single CdSe NWs before and after CE, with different ratios of the CdSe and Cu_2−*x*_Se length sections between the probing electrodes. In this approach, we used a planar device configuration with fixed on-chip electrodes, resulting in a device structure in which a single wire is connected by two electrodes, as shown in Fig. 6a. The inset in Fig. 6a illustrates the NW with a CdSe/Cu_2−*x*_Se heterojunction between the electrodes that was obtained by our masked CE procedure. Fig. 6b shows the NW in the electrode gap at higher magnification, together with EDS maps that were recorded after all measurements. Here the heterojunction interface clearly falls within the electrode gap, with the left section of the NW consisting of CdSe, while the right section is composed of Cu_2−*x*_Se. Representative current–voltage curves of a single NW before and after masked CE are displayed in Fig. 6c. In the transport experiments, the contact at the CdSe section was biased, and the Cu_2−*x*_Se side was connected to ground. The CdSe NW shows a very low dark current (black trace), and a photocurrent that first rises with the applied bias, eventually saturating to an almost constant value. This trend has already been observed in our previous report on CdSe NWs, and it was attributed to the saturation of the drift velocity of electrons by various scattering mechanisms under high electric field^42^. The partially exchanged CdSe/Cu_2−*x*_Se NW in Fig. 6c manifests higher conductance compared to the starting CdSe NW. Interestingly, the photoconductivity is also strongly enhanced in the heterojunction NW, although no photocurrent was observed from fully transformed Cu_2−*x*_Se NWs (see Supplementary Notes 6, Supplementary Figure 9). Furthermore, the photo-IVs of the heterojunction NWs are strongly asymmetric (as can be expected from the band structure in Fig. 5c), and the photocurrent shows no saturation. The asymmetry of the photo-IVs can also result from the different contacts formed by the Al electrodes to the CdSe and Cu_2_Se nanowire sections. Al with a work function at 4.08 eV^43^ aligns well with the Fermi level that we obtained for the Cu_2−*x*_Se NWs, but falls within the band gap of the CdSe NWs. Therefore, we can expect a quasi ohmic contact on the Cu_2_Se side, and a Schottky contact on the CdSe side.

Figure 6d, e compare the dark current of single CdSe and Cu_2−*x*_Se NWs with heterojunction NWs, in which different length sections between the electrodes have been transformed from CdSe to Cu_2−*x*_Se by masked CE. The conductance dramatically increases with increasing the length of the Cu_2−*x*_Se segment. This can be straightforwardly related to the higher conductivity of Cu_2−*x*_Se, which is characterized by free carriers originated by the high density of Cu vacancies, as well established for this system^36,44,45^. The overall conductance of the wires as a function of the length of the Cu_2−*x*_Se section is displayed in Fig. 6e, manifesting a non-linear increase with Cu_2−*x*_Se section length. Here the conductance *σ* varies over 6 orders of magnitude with different Cu_2−*x*_Se section lengths. In Fig. 6e, the conductance of the heterojunction nanowires deviates from what would be expected from two ohmic resistors in series with the respective conductivities obtained at −3 V (dotted line in Fig. 6e), which can be attributed to the non-linear IVs of the CdSe NWs. Due to the much higher conductance of the Cu_2−*x*_Se section, the applied bias drops almost completely on the CdSe section, which increases the effective field strength (V/cm) for the shorter sections dramatically.

In conclusion, we demonstrated that single CdSe NWs can be patterned in laterally structured CdSe/Cu_2_Se heterojunctions by masked CE. The heterojunction interface is nearly atomically sharp due to hindered axial cation diffusion and the high energy barrier for exchange via radial diffusion in the masked regions. These insights can be of great interest for CE on single nanostructures with other shapes and composition such as, for example, nanosheets. The masked CE can be performed on-chip and enables transformations of already fabricated devices, which can be very useful for extending the applicability of existing device concepts.

## Methods

### CdSe nanowire synthesis and cation exchange reaction

The NWs were synthesized following the protocol as described in former works^42,46^. The major detail added in the present reaction scheme was the careful removal of all excess humidity by applying a vacuum at various stages of the reaction. Syntheses based on phosphonic acids tend to form gels in the final product. The gel, being a bulky organic residue, could by itself inhibit the cation exchange reactions on the substrates. Therefore, it is of utmost importance for the described experiments to minimize the amount of gel present in the solution of NWs. Most often, the gel formation is triggered by the addition of methanol while the solution is still cooling down. Apparently, also the water formed when CdO decomposes at ca. 300 °C could remain in traces in the solution and trigger the gel formation in a similar way. Therefore, the solution was first degassed as usual at ca. 120 °C, then heated until the red color of the CdO faded, and then cooled down again to ca. 150 °C. At this stage, the solution was degassed for ca. 30 min, after which the solution was heated to the required temperature and the growth of the NWs was started by the injection of Bi and Se as reported in literature^42,46^. Additionally, vacuum could also be applied after the reaction when the solution is cooled to below 150 °C. The CE reaction was performed by the immersion of the substrate in a methanolic solution (4 mL) of Cu^+^ cations (2 mM solution of Tetrakis(acetotnitrole) copper (I) hexafluorophosphate in methanol, 75 mg/mL) for 5 min. The reaction was stopped by washing the samples in methanol and drying them under nitrogen flow.

### Device fabrication and optoelectrical characterization

CdSe NWs suspended in toluene were spin-cast on a SiO_2_/Si substrate pre-patterned with Ti/Au markers. The position of single NWs was localized with respect to the position of the Au markers under a scanning electron microscope. The electrodes were defined by electron-beam lithography followed by evaporation of 4/50 nm Titanium/Aluminum for the contacts. The current–voltage (IV) characteristics were studied under vacuum conditions using a cryogenic probe station system (Janis Research). A microscope lamp (Schott EKE AEI) was used as an illumination source. After this first measurement series, partial CE was performed on the contacted nanowires to compare the electrical properties of the same NW before and after CE. To this aim, sections of the NWs in between the electrodes (that partially overlap with one electrical contact) were exposed with an electron-beam using a Raith 150-two lithography system. We used an acceleration voltage of 10 kV and an optimized exposure dose of 400 mC/cm^2^ to cross-link the organic surface ligands. We then performed the CE as described above, and the samples were transferred back into the probe-station where their electrical properties were measured again.

### Transmission electron microscopy

Samples were prepared by dropping dilute solutions onto ultrathin carbon/holey carbon coated 400 mesh gold grids for e-beam patterning, followed by cation exchange. Afterwards, high-resolution TEM (HRTEM) of the NWs was performed with a JEOL JEM-2200FS microscope equipped with a 200 kV field emission gun, a CEOS spherical aberration corrector for the objective lens and an in-column image filter (Ω-type). Energy dispersive X-ray spectroscopy (EDS) elemental maps of the NWs were acquired by a Bruker Quantax 400 system with a 60 mm^2^ XFlash 6 T silicon drift detector (SDD) mounted on the same microscope.

### Computational modeling

First principle calculations were performed using the generalized gradient approximation with PBEsol exchange correlation functional^47^, as implemented in the pwscf code, within Quantum Espresso (QE) package^48^. We employed the PAW formalism^49^ and datasets from the website www.quantum-espresso.org (Table S1). The Nudged Elastic Band (NEB) calculations to determine the reaction energy barriers were performed using the neb.x code included in the QE package. Further details are given in the Supplementary Information.

### X-ray and ultraviolet photoelectron spectroscopy

For XPS and UPS, the nanowire samples were drop-cast on gold-coated (50 nm) silicon wafers, and measurements were performed using a Kratos Axis Ultra DLD spectrometer. Photoelectrons were detected at a takeoff angle *Φ* = 0° with respect to the surface normal. The pressure in the analysis chamber was maintained below 5 × 10^−9^ Torr for data acquisition. The data was converted to VAMAS format and processed using CasaXPS software. An energy scale calibration was performed setting the Au 4f_7/2_ peak at a fixed binding energy of 84.0 eV. XPS was conducted with a monochromatic Al Kα source (15 kV, 20 mA) and the spectra were acquired at a pass energy of 10 eV with steps of 0.1 eV. UPS was conducted with a He I (21.22 eV) discharge lamp and the spectra were acquired at a pass energy of 5 eV with steps of 0.025 eV. The position of the Fermi level with respect to vacuum level is measured from the threshold energy for the emission of secondary electrons during He I excitation. A bias of −9.0 V was applied to the sample in order to precisely determine the low kinetic energy cutoff, as discussed in ref. ^50^. Then, the position of the VBM vs. vacuum level was estimated by the energy difference of the onset of the spectrum at low binding energy with respect Fermi level, according to the graphical method used in ref. ^51^.

### Data availability

The authors declare that the main data supporting the findings of this study are available within the article and its Supplementary Information files. Extra data are available from the corresponding authors upon request.

## Electronic supplementary material

Supplementary Information
