# Supplementary material for: Lateral epitaxial heterojunctions in single nanowires fabricated by masked cation exchange
Source: Nat Commun. 2018 Feb 6;9:505. doi: 10.1038/s41467-018-02878-w (PMC5802801; doi:10.1038/s41467-018-02878-w)
Supplement: Supplementary file 1 — Supplementary Information [file 41467_2018_2878_MOESM1_ESM.pdf]

Supplementary Notes 1-6, with Supplementary Figures and Supplementary Tables therein

Supplementary Notes 1: High-Resolution Transmission Electron Microscopy

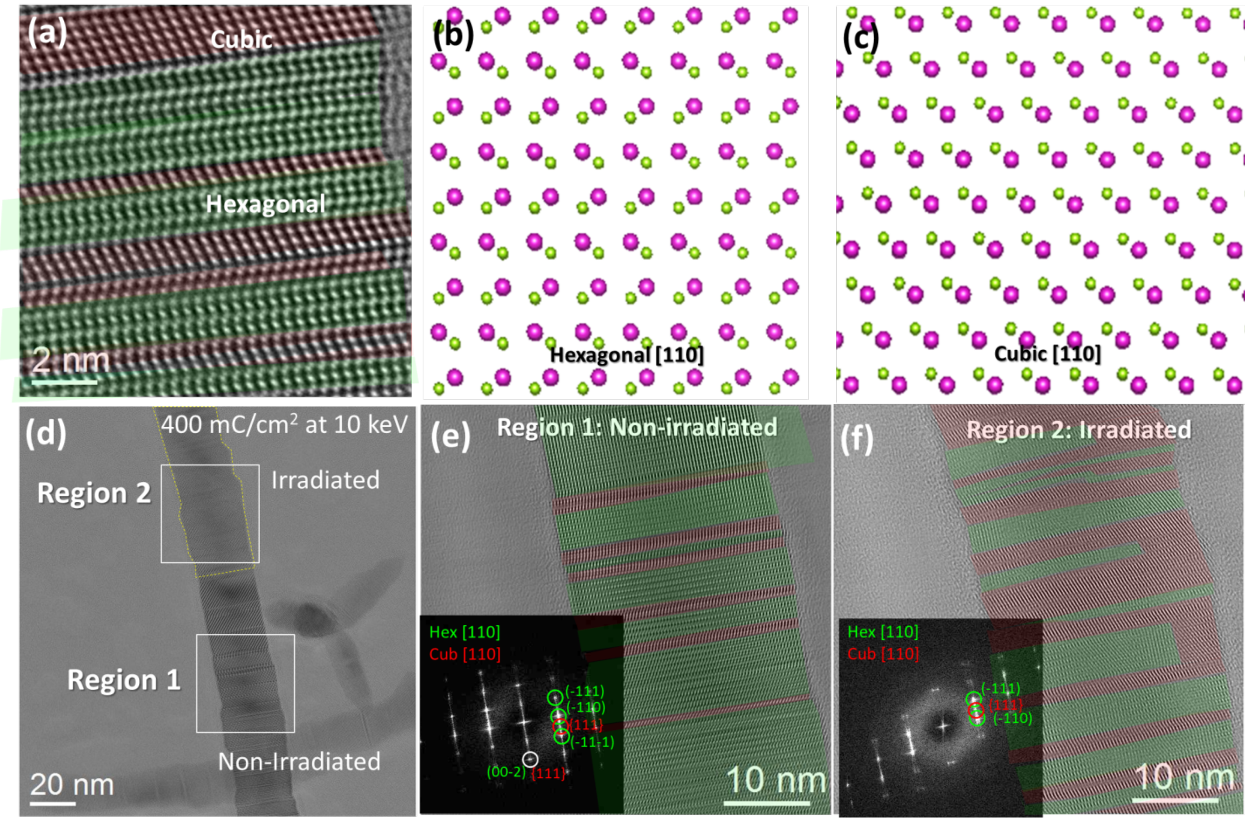

Supplementary Figure 1. *(a) HRTEM image of a CdSe wire with both hexagonal phase domains (green color) and cubic phase domains (red color); (b, c) Crystal model of hexagonal (b) and cubic (c) CdSe in the [110] zone axis; (d) TEM image of a wire in which region 2 has been exposed to the electron-beam at 10 kV with a high dose of 400 mC/cm*^{2}*; (e, f) Magnified view of the non-irradiated and irradiated regions shown in (d). Cubic sections have been colored in red, hexagonal ones in green.*

Supplementary Figure 1 (a) shows a NW oriented in the [110] direction with sections of both hexagonal and cubic phases alternatively distributed along the wire. Here, domains in green are in hexagonal phase (crystal model shown in Supplementary Figure 1 (b)) and the red colored domains are in cubic phase (crystal model shown in Supplementary Figure 1 (c)).

We have investigated the effect of e-beam irradiation on the crystallinity of the CdSe nanowire. Supplementary Figure 1 (d) shows a HRTEM image of a wire in which only the upper part has been irradiated with electrons at an acceleration voltage of 10kV with a dose of 400 mC/cm^2. As discussed above, the pristine region contains sections of hexagonal and cubic phase (see Supplementary Figure 1 (e)), of which the majority are in the hexagonal phase (green). In comparison, after electron beam irradiation, the fraction of sections in the cubic phase (red) increases (see Supplementary Figure 1(f)).

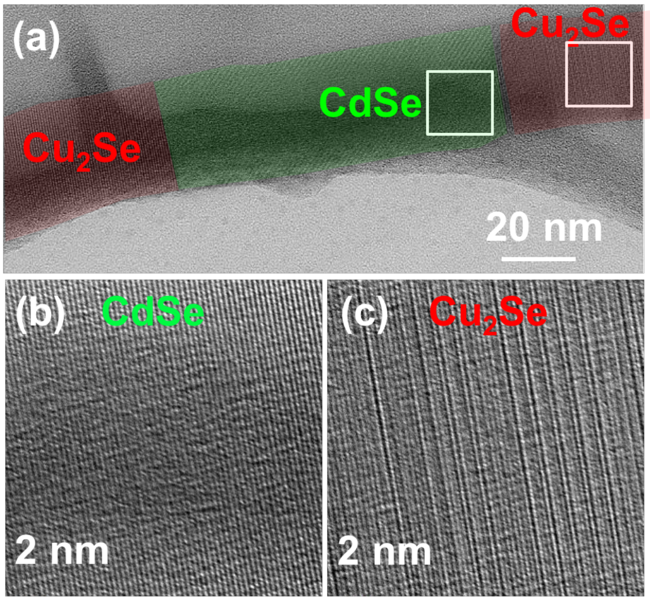

Supplementary Figure 2. *HRTEM image of a CdSe/Cu_2Se heterojunction (a) and magnified views of the CdSe (b) and Cu_2Se (c) regions that are marked with boxes in (a). The magnified views show that the defects in the masked CdSe region have been removed by electron irradiation, while the Cu_2Se region is rich in stacking faults.*

Supplementary Notes 2: **CE experiments with varying cation concentration.**

We have performed CE experiments with varying cation concentration to obtain data for the initial process. These experiments have been carried out in solution by adding different amount of Cu^+ ions. We divide our data in two regimes with low (1) and high (2) Cu^+ concentration. A detailed HRTEM and STEM-EDS analysis gave the following results:

(1) Cu^+/Cd^{2+}=0.5 to 1: In some of the wires, only a very small amount of Cu^+ is found distributed all along the wire (Supplementary Figure 3 (a)). For other wires, completely exchanged segments were observed (Supplementary Figure 3 (b, c));
(2) Cu^+/Cd^{2+}=2 to 4: all the wires were exchanged (Supplementary Figure 3 (d)).

The above results indicate that the transformation in the non-irradiated regions takes place in a way that a short Cu_2Se section quickly forms and then it expands throughout the whole wire.

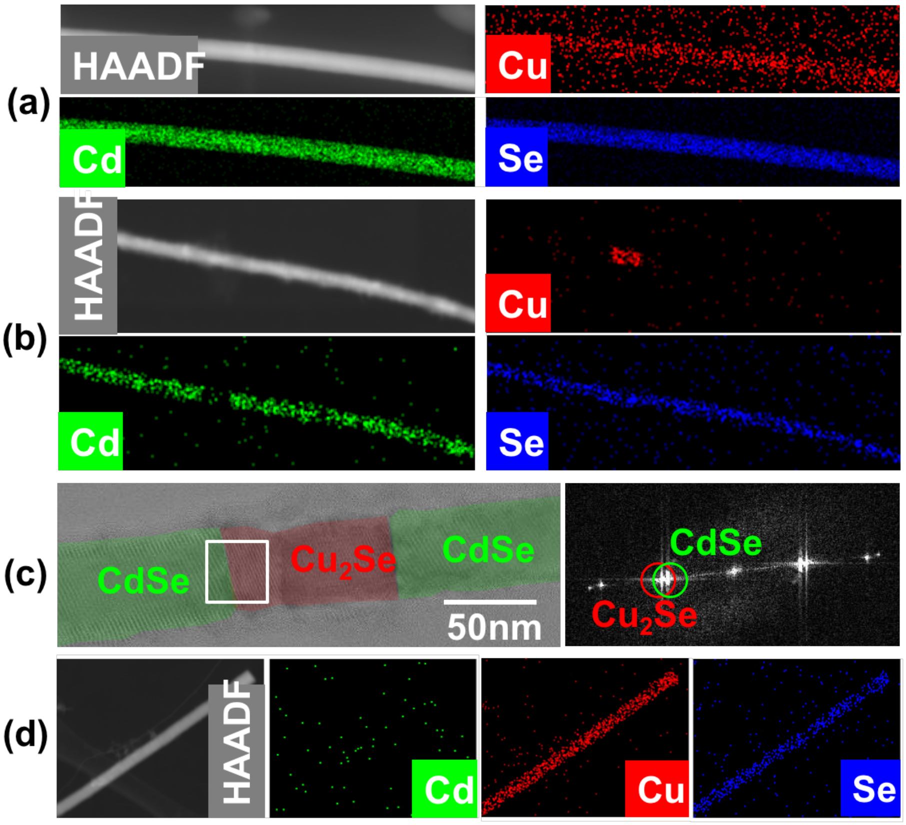

Supplementary Figure 3. *(a) HAADF image and elemental maps of a CdSe NW from a sample that underwent reaction with Cu^+ ions, at a Cu^+/Cd^{2+} ratio equal to 1. In this case, Cu atoms are distributed along the wire (Cu:Cd:Se=3%:45%:52%); (b) HAADF image and elemental maps of another CdSe NW, but from the same sample as (a). From the maps, it can be seen that, in this NW, a CdSe segment has been exchanged to Cu_2Se; (c) Left: The HRTEM image of the region around the exchanged segment shown in (b). Right: FFT corresponding to the boxed area in the left panel; (d) HAADF image and elemental maps of a CdSe NW from a sample that underwent reaction with Cu^+ ions, at a Cu^+/Cd^{2+} ratio equal to 2. In this case, the wire has been entirely cation exchanged to Cu_2Se.*

Supplementary Notes 3: X-Ray Photoelectron Spectroscopy on Cu_2Se NWs

X-ray photoelectron spectroscopy (XPS) was performed on (not masked) NWs right after cation-exchange (Supplementary Figure 4a,b) and after exposure to air for 24h (Supplementary Figure 4c,d). After cation-exchange, quantitative analysis gives a Cu:Se ratio of 2.0:1, which suggests that cation-exchange yields fully stoichiometric Cu_2Se NWs. As expected, the Cu 2p spectrum (Supplementary Fig. 4a) presents only two peaks for Cu $2p_{3/2}$ and Cu $2p_{1/2}$ at binding energies BE = 932.6 eV +/- 0.1 eV and BE = 952.4 eV +/- 0.1 eV, typical of Cu(I).[1] The oxidation state of +1 is also confirmed by X-ray-excited Auger Electron Spectroscopy (XAES; Supplementary Fig. 5). Likewise, Se 3d spectrum (Supplementary Fig. 4b) can be fitted with Se $3d_{5/2}$ and Se $3d_{3/2}$ at BE = 54.0 eV +/- 0.1 eV and BE = 54.9 eV +/- 0.1 eV typical of Se^{-2} in Cu_2Se.[1] We also note a slight contribution from SeO_x species at higher BE. As has also been noted by others,[1] after exposure to air Cu_2Se nanocrystals oxidize (Supplementary Fig .4c,d). In Supplementary Figure 4c, Cu(II) species are

clearly identified by the second Cu 2p doublet at BE = 934.6 eV +/- 0.1 eV (Cu(II) $2p_{3/2}$) and BE = 954.4 eV +/- 0.1 eV (Cu(II) $2p_{1/2}$) as well as by the satellite peaks at higher BE. Se 3d spectrum (Supplementary Figure 4d) shows an increase of SeO_x species but most importantly the presence of a new contribution with $Se3d_{5/2}$ at BE = 54.5 eV, which translates the formation of Se-Se bonds in substoichiometric $Cu_{2-x}Se$.[1]

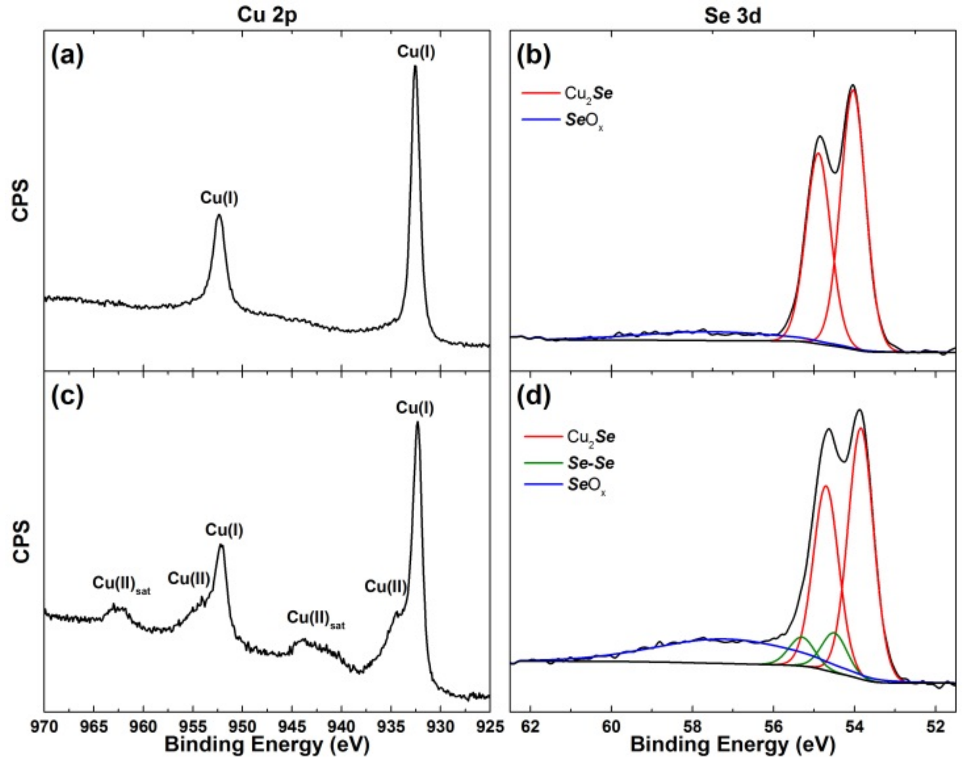

Supplementary Figure 4. *XPS characterization of NWs right after cation-exchange (a,b) and after 24h exposure to air (c,d).*

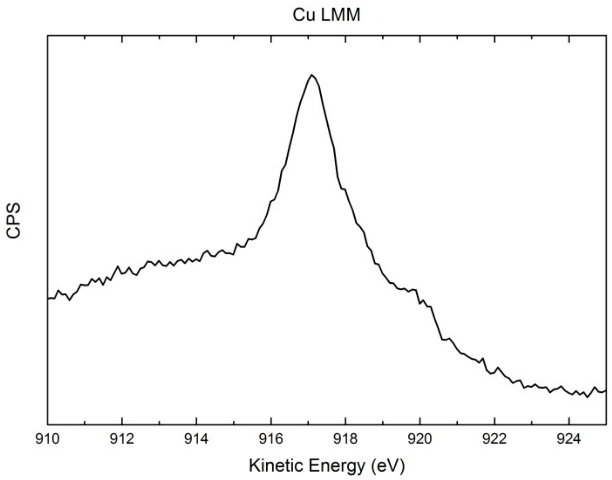

Supplementary Figure 5. *XAES Cu LMM signal of NWs after cation exchange. The peak position at a kinetic energy KE = 917 eV is typical of Cu(I).*[2]

Supplementary Notes 4: Computational Modeling

a) Technical details

First principles calculations were performed using the generalized gradient approximation with PBEsol exchange correlation functional, [3] as implemented in the Quantum Espresso (QE) package.[4] We employed the PAW formalism [5] and datasets [8] available on the website www.quantum-espresso.org (Supplementary Table 1).

The Nudged Elastic Band (NEB) calculations to determine the diffusion energy barriers were performed using the neb.x program from the QE package. For CdSe, we used 72-ion supercells composed of 3x3x2 four-atom CdSe unit cells, with a single Cu impurity. The Gamma point branch of the algorithms was used. The energy cutoff was chosen to be 30 Ry. Unit cell vectors were fixed along the NEB to those corresponding to the full relaxation of the initial configuration. Twelve images including the initial and the final ones were used to sample the NEB. The threshold of the force orthogonal to the path was set to 0.1 eV/A, and energy convergence threshold of 10^{-8} Hartree was used. The variable elastic constants (k_min and k_max) between 0.2 and 0.3 Bohr were used and climbing image was on in all calculations.

In the estimation of the lower bound on the CE barrier we used a supercell containing the interface between antifluorite Cu2Se and sphalerite CdSe structures. A cubic supercell, shown in Supplementary Fig. 6, was used. It contains 32 Se, 32 Cu, and 19 Cd atoms, plus a Cu and a Cd impurity. The antifluorite and sphalerite structures were matched along the (111) plane of the conventional cubic unit cell, and the impurities were placed in a void in the middle layer of structure to mimick isolated impurities as well as possible. A 4x4x4 Gamma-centered k-point grid, an energy cutoff of 30 Ry and a Gaussian smearing of 0.01 Ry for Brillouin zone integration were used. We performed a full relaxation of the structures, preceded by volume optimizations, in both settings, until the total force on ions reached below 0.0015 Ry/Bohr.

b) Estimation of the diffusion barriers of Cu^+ in the wurtzite CdSe

First, we analyze the CE in the initial stage in the non-irradiated part of the nanowire, we compare the diffusion rates of Cu^+ cations in radial and axial directions of NW. The hexagonal **c** axis of CdSe structure is along the axis of the NW.

We determine the diffusion rate of the impurity according to the transition state theory [6], that gives an Arrhenius-like dependence of the reaction rate on the free energy difference between the initial configuration and the transition state, defined as the saddle point on the reaction path. Within this scheme, the particle follows the so-called minimum energy path (MEP), which can be defined as a union of steepest descent paths from the saddle point to the minima. The MEP has the property that any point on the path is at an energy minimum in all directions perpendicular to the path [7]. This allows a systematic search for the MEP. We used the Nudged Elastic Band Method [7] implemented in the neb.x program within the Quantum Espresso package to compute the MEP and the reaction rates.

In our quest for the local minima representing metastable states involved in diffusion, we identify two interstitial equilibrium positions:

A: the impurity lies in the middle of the hexagonal ring in the Cartesian XZ and YZ planes,

B: the impurity lies in the middle of the hexagonal ring in the Cartesian XY plane.

There are three possible different diffusion paths in each direction, namely AA, BB and ABAB. We find that the lowest energy diffusion paths for the Cu+ cation link the A and B positions. Through the lowest energy diffusion in radial direction, the lowest energy barrier is 170 meV (labelled as AB). Through the axial direction, we find that the lowest energy barrier is 350 meV (labelled as AB2). The latter is about 2 times larger than the former, so the radial diffusion is roughly 8 times faster than the axial. In addition, we identify two more diffusion paths in axial direction, connecting adjacent A (axial AA) and B points (axial BB). See Supplementary Figure 6 for structural details of the four paths, and Fig. 3a in the main text for plots of the diffusion barriers.

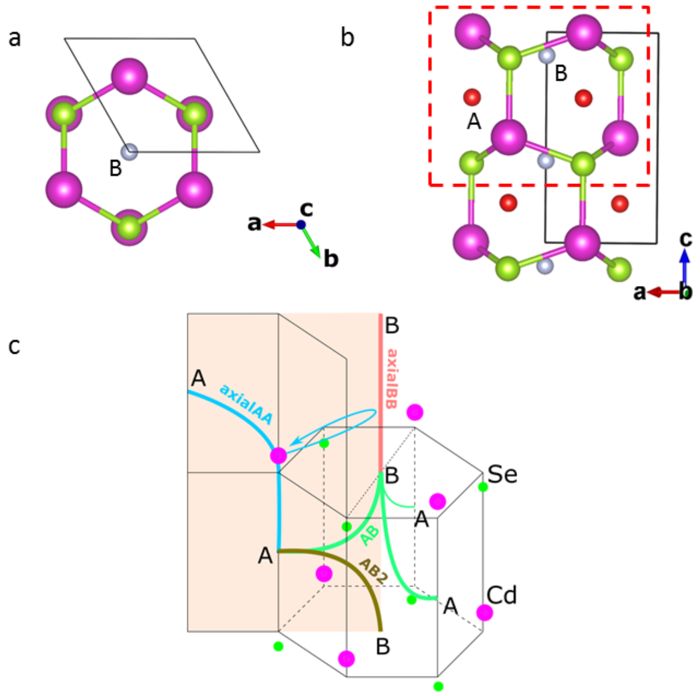

Supplementary Figure 6. *Scheme showing the hexagonal prism-shaped void selected within the CdSe structure to represent the four diffusion paths studied. The local minima, labelled as A and B in the text, are also shown. In (a) and (b) the projections on the planes perpendicular to the c and b axes are given, respectively, alongside the unit cell of CdSe. The A type impurity-sites are represented with red spheres and B type impurity-sites with grey spheres. In (c) a representation of this void, limited by a red dashed border rectangle in (b), is drawn, showing the four diffusion paths studied. Cd atoms are represented in magenta and Se atoms in green. The low-energy barrier A-B (labelled as AB), high-energy barrier A-B (labelled as AB2), axial A-A and axial B-B are drawn in green, brown, blue and orange, respectively. The radial diffusion is dominated by successive AB diffusions, while the lowest energy axial diffusion path consists of alternating AB and AB2 diffusions laying on the same plane, indicated by the orange rectangle.*

__Supplementary Table 1.__ Energy barriers of the four diffusion paths, in meV units.

**Path**	**Energy (meV)**
**A-B (AB)**	**0.17**
**A-B (AB2)**	**0.35**
**A-A (axial)**	**0.39**
**B-B (axial)**	**0.48**

c) Calculations on the interface diffusion

The calculations were performed on four 3*3*3 supercells, as described in the main text. The interstitial defects and the vacancies were separated as much as possible within the cells. The energy cutoff of 40 Ry was used for the wavefunctions, 320 Ry for the charge density. The occupations were computed using Marzari-Vanderbilt smearing of 0.01 Ry. A shifted 2x2x2 k-point grid was used in the Brillouin zone integration. We performed a full relaxation of the structures, preceded by volume optimizations, in both settings, until the sum of absolute values of all forces was below 0.002 Ry/Bohr.

For the diffusion of Cu^{+} cations in the irradiated area, we approximate the barriers by those we obtained for the radial diffusion in Wurtzite (as calculated in section (a) above). This may be an appropriate approximation since the pathways in sphalerite in (1, 0.5, 0.5) direction and in wurtzite (-1,1,0) direction are very similar, as illustrated in Supplementary Figure 7 that shows the views along these two directions in (b) and (a), respectively.

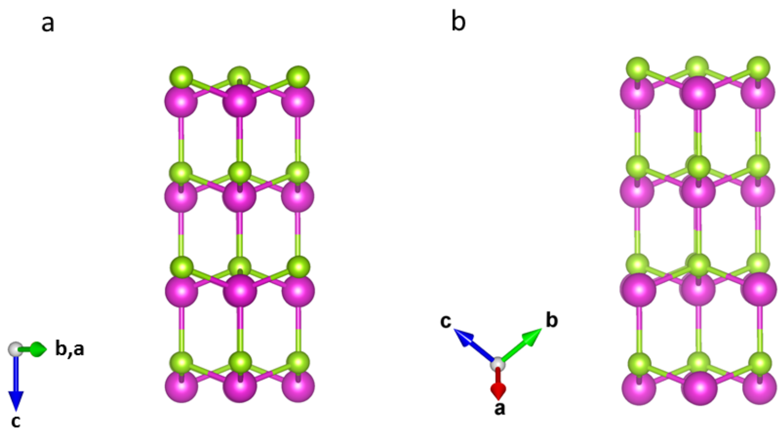

***Supplementary Figure 7**. (a) hexagonal wurtzite CdSe structure projected along the (-1,1,0)* radial *direction and (b) the sphalerite CdSe in the (1, 0.5, 0.5) direction of the conventional cubic cell.*

Supplementary Notes 5: Band scheme of $Cu_{2-x}Se$/CdSe heterojunction

We have performed Ultraviolet Photoelectron Spectroscopy (UPS) on the original CdSe NWS, as well as on the cation-exchanged, and oxidized, $Cu_{2-x}Se$ NWs. This technique allows to determine the energy difference between vacuum and Fermi level, and between the valence band and Fermi level. We note that due to the soft increase in signal in (b) the evaluation of the valence band to Fermi energy difference is less precise. This is a well-known limitation of this technique, as for example reported in Supplementary ref. 9.

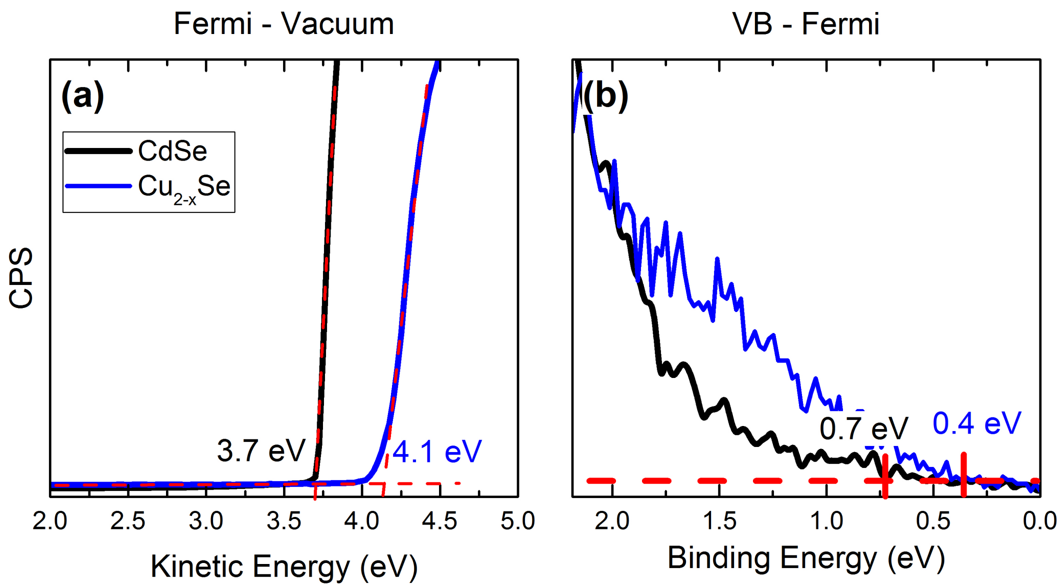

Supplementary Figure 8. *Magnified sections of the UPS spectrum plotted as kinetic (a) and binding (b) energy that allow to estimate the Fermi level at 4.1 eV below vacuum for the* $Cu_{2-x}Se$ *NW, and at 3.7 eV for the CdSe NWs, and the top of the valence band to Fermi level energy to 0.4 eV and 0.7 eV for* $Cu_{2-x}Se$ *and CdSe NWs, respectively.*

Supplementary Notes 6: Additional transport data

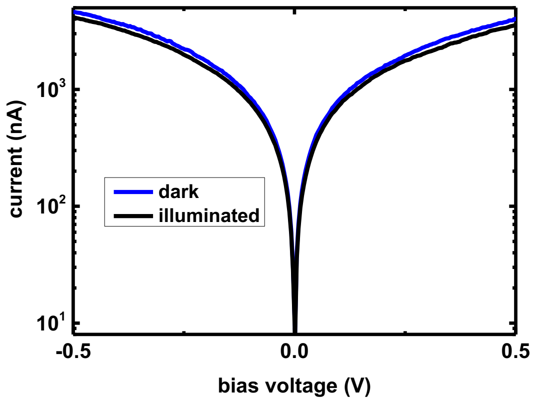

Supplementary Figure 9. *Dark and photo IV characteristics of a single* $Cu_{2-x}Se$ *NW.*

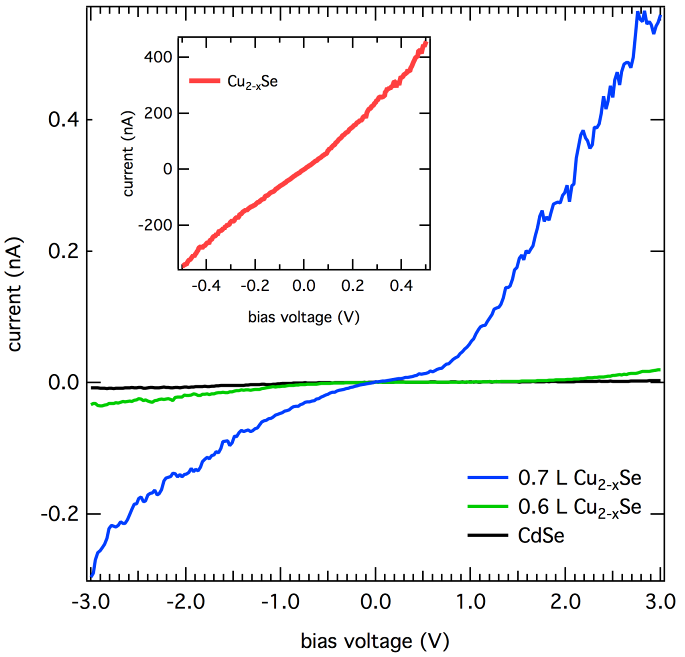

Supplementary Figure 10. *Linear plots of the data shown in Figure 5d of the main text.*

Supplementary References

1. Riha, S. C.; Johnson, D. C.; Prieto, A. L., Cu_2Se nanoparticles with tunable electronic properties due to a controlled solid-state phase transition driven by copper oxidation and cationic conduction. *J. Am. Chem. Soc.* **2011,** *133*, 1383.
2. Biesinger, M. C.; Lau, L. W. M.; Gerson, A. R.; Smart, R. S. C., Resolving surface chemical states in XPS analysis of first row transition metals, oxides and hydroxides: Sc, Ti, V, Cu and Zn. *Appl. Surf. Sci.* **2010,** *257*, 887.
3. Perdew, J. P.; Ruzsinszky, A.; Csonka, G. I.; Vydrov, O. A.; Scuseria, G. E.; Constantin, L. A.; Zhou, X.; Burke, K., Restoring the density-gradient expansion for exchange in solids and surfaces. *Phys. Rev. Lett.* **2008,** *100*, 136406.
4. Giannozzi, P.; Baroni, S.; Bonini, N.; Calandra, M.; Car, R.; Cavazzoni, C.; Ceresoli, D.; Chiarotti, G. L.; Cococcioni, M.; Dabo, I., Quantum espresso: A modular and open-source software project for quantum simulations of materials. *J. Phys. Cond. Matter* **2009,** *21*, 395502.
5. Blöchl, P. E., Projector augmented-wave method. *Phys. Rev. B* **1994,** *50*, 17953.
6. Eyring, H., The activated complex and the absolute rate of chemical reactions. *Chem. Rev.* **1935,** *17*, 65.
7. Mills, G.; Jónsson, H.; Schenter, G. K., Reversible work transition state theory: Application to dissociative adsorption of hydrogen. *Surf. Sci.* **1995,** *324*, 305-337.
8. Kucukbenli, E., Monni, M., Adetunji, BI., Ge, X., Adebayo, G. A., Marzari, N., de Gironcoli, S., Dal Corso, A., Projector augmented-wave and all-electron calculations across the periodic table: a comparison of structural and energetic properties. arXiv:1404.3015
9. Hoye, R. L. Z.; Schulz, P.; Schelhas, L. T.; Holder, A. M.; Stone, K. H.; Perkins, J. D.; Vigil-Fowler, D.; Siol, S.; Scanlon, D. O.; Zakutayev, A.; Walsh, A.; Smith, I. C.; Melot, B. C.; Kurchin, R. C.; Wang, Y.; Shi, J.; Marques, F. C.; Berry, J. J.; Tumas, W.; Lany, S.; Stevanović, V.; Toney, M. F.; Buonassisi, T., Perovskite-inspired photovoltaic materials: toward best practices in materials characterization and calculations. *Chem. Mater.* **2017,** *29*, 1964.
